# Lactic Acid Bacteria and Aging: Unraveling the Interplay for Healthy Longevity

**DOI:** 10.14336/AD.2023.0926

**Published:** 2024-08-01

**Authors:** Rui Liu, Bo Sun

**Affiliations:** ^1^School of Food Engineering, Ludong University, Yantai, Shandong 264025, China; ^2^State Key Laboratory of Digital Medical Engineering, Southeast University, Nanjing 210096, China

**Keywords:** lactic acid bacteria, aging, gut health, immune function, oxidative stress

## Abstract

Lactic Acid Bacteria (LAB) are beneficial microorganisms widely utilized in food fermentation processes and as probiotic supplements. They offer multifarious health benefits, including enhancing digestion, strengthening immune mechanisms, and mitigating inflammation. Recent studies suggest that LAB might be instrumental in the anti-aging domain, modulating key molecular pathways involved in the aging continuum, such as IL-13, TNF-α, mTOR, IFN-γ, TGF-β, AMPK, and GABA. The TLR family, particularly TLR2, appears pivotal during the primary cellular interactions with bacteria and their byproducts. Concurrently, the Sirtuin family, predominantly Sirtuin-1, plays diverse roles upon cellular stimuli by bacterial components. The potential anti-aging benefits postulated include restoring gut balance, enhancing antioxidant potential, and fortifying cognitive and mental faculties. However, the current body of evidence is still embryonic and calls for expansive human trials and deeper mechanistic analyses. The safety and optimal consumption metrics for LAB also warrant rigorous evaluation. Future research trajectories should identify specific LAB strains with potent anti-aging properties and unravel the underlying biological pathways. Given the promising implications, LAB strains stand as potential dietary contenders to foster healthy aging and enrich the quality of life among the elderly population.

## 1. Introduction

Aging is a complex and multifaceted process influenced by various factors, including genetics, lifestyle choices, and environmental exposures [1]. Genetic factors play a significant role in determining the rate of aging and overall lifespan. However, lifestyle choices, such as diet, exercise, and smoking, can also profoundly impact the aging process. Additionally, environmental exposures, including pollution and radiation, can contribute to the acceleration of aging [2]. The consequences of aging are far-reaching, leading to a decline in physical function, such as reduced mobility and strength, as well as an increased risk of chronic diseases, including cancer, heart disease, and neurodegenerative disorders like Alzheimer's disease [3]. These age-related conditions significantly affect an individual's overall health and quality of life.

Lactic Acid Bacteria (LAB) represents a group of bacteria commonly found in fermented foods, including yogurt, kefir, and sauerkraut. Extensive genome-wide analysis has demonstrated the presence of closely related LAB strains in both food and gut environments, providing compelling evidence of the connection between fermented foods and the gut microbiome [4]. The gut microbiome consists of trillions of microorganisms residing in the digestive tract, playing a vital role in digestion, nutrient absorption, and immune function [5]. Consuming foods rich in LAB has been shown to improve gut health by increasing the abundance of beneficial bacteria in the gut.

Studies indicated LAB, as a prominent component of the gut microbiota, has the potential to impact aging through their effects on gut health, immune function, and the gut-brain axis [6-8]. Consuming LAB-rich foods or utilizing LAB supplementation may offer a promising adjunctive approach to support healthy aging and improve overall well-being. The intricate mechanisms underlying the relationship between LAB and aging will be explored and summarized in this review and try to provide valuable insights and perspectives on the topic.

## 2. Potential Mechanisms of Anti-aging Effects

Molecular mechanisms of aging include genomic instability, telomere dysfunction, epigenetic alterations, loss of proteostasis, compromise of autophagy, mitochondrial dysfunction, cellular senescence, and stem cell exhaustion [9-12]. Gut microbiota such as LAB play a significant role in maintaining overall health and may impact the aging process [13, 14]. In this part, we explored the possible mechanisms through which LAB may influence aging, including their impact on gut homeostasis, immune function, inflammation, oxidative stress, and the gut-brain axis. Understanding the interplay between LAB and aging processes could pave the way for innovative strategies to promote healthy aging and improve the quality of life for the elderly.

### 2.1. LAB Modulation Gut Barrier Integrity for aging mitigation

The gut plays a pivotal role in nutrient absorption and the regulation of harmful substances. The gut barrier is a fundamental component in regulating various physiological processes, acting as both a physical and functional separator between the host's internal milieu and the external environment. A compromised intestinal barrier enables the passage of microbes and unwanted antigens through the epithelium, triggering systemic endotoxemia-associated inflammation [15-17]. This inflammatory process is implicated in various diseases, spanning metabolic syndrome, chronic organ dysfunction, and age-related ailments like neurodegenerative disorders and cancer.

Compelling evidence highlights the pivotal involvement of aging-related genes, particularly SIRTs1, 3, and 6, in safeguarding gut barrier integrity and modulating gut inflammation, underscoring their significance in the context of gut health and disease pathogenesis [9, 18, 19]. Understanding the intricacies of age-related gut barrier changes is paramount for comprehending their broader impact on human health. This knowledge can inform the development of targeted interventions aimed at mitigating the adverse effects of a compromised gut barrier in the context of aging.

Based on investigating the unique influence of LABs on goblet cell-associated genes essential for mucus production and function, such as MUC2, TFF3, RETNLB, CHST5, and GAL3ST2, various LABs modulate goblet cells with distinctive effects contingent on species and specific strains. During TNF-α challenges, S. thermophilus CCFM218 and L. rhamnosus CCFM237 notably enhanced GAL3ST2 gene expression. Conversely, with IL-13 exposure, select LAB strains like L. plantarum CCFM734, S. thermophilus CCFM218, and L. rhamnosus CCFM237 inhibited IL-13-induced upregulation of TFF3 and RETNLB expression [22]. Within the same study, the effects of heat-killed bacteria and bacterial cell-free culture medium (CM) on goblet cell-related gene expression were examined. Intriguingly, this investigation revealed that heat-killed LAB did not elicit any noticeable alterations in gene expression. In contrast, CM derived from specific LAB strains demonstrated substantial regulation of genes linked to mucus function, suggesting the production of specialized bacterial factors influencing goblet cell activity. Notably, CM from L. fermentum CCFM787 significantly augmented gene transcription, signifying the presence of bioactive agents with the potential to modulate goblet cells [22]. These findings align with our previous research, which characterized bioactive compounds, such as cyclic dipeptides, showcasing their antimicrobial properties [23, 24].

In the context of host-microbe interactions, Toll-like receptor 2 (TLR2) plays a pivotal role in recognizing peptidoglycan, a major constituent of gram-positive bacteria, including those belonging to the Lactobacillus genus [25]. Specifically, Lactobacillus casei 431 has been observed to engage epithelial cells through TLR2, fostering interactions that result in an elevated presence of CD-206 and TLR2 receptors among gut-associated immune cells [26]. Furthermore, TLR2 signaling activation by Lactobacillus plantarum has been documented, subsequently implicating the activation of protein kinase C-α and -δ in the modulation of tight junctions and epithelial permeability [27].

In a parallel study, an intentional induction of dysbiosis in the rat's intestinal tract was undertaken [28]. Remarkably, when these rats were administered a probiotic mixture comprising four distinct strains, namely Bifidobacterium breve DM8310, Lactobacillus acidophilus DM8302, Lactobacillus casei DM8121, and Streptococcus thermophilus DM8309, a substantial enhancement in mucosal barrier integrity was observed. Additionally, this therapeutic intervention resulted in significant reductions in the concentrations of proinflammatory factors and cytokines, accompanied by a diminished influx of neutrophils. These discernible outcomes are intricately associated with the restoration of intestinal microbial equilibrium and concurrent modifications in the signaling pathways governed by TLR2 and TLR4. These findings underscore the multifaceted role of probiotics in influencing host-microbe interactions and intestinal homeostasis.

### 2.2 LAB Modulation of Gut Microbiota Composition for Ageing Prevention

Aging is accompanied by notable changes in the composition and diversity of the gut microbiota, a phenomenon referred to as dysbiosis [20-22]. The gut microbiota composition is subject to dynamic changes influenced by a multitude of factors, such as dietary patterns, advancing age, genetic predisposition, and lifestyle choices [23-26]. Emerging evidence suggests that LAB plays a pivotal role in maintaining gut homeostasis by actively contributing to the intricate balance of microbial communities.

Numerous studies have explored the impact of LAB supplementation on gut microbiota composition, with a specific focus on elderly individuals [27, 28]. Notably, a study published in the esteemed journal Nature demonstrated that LAB supplementation in elderly subjects resulted in a significant increase in the abundance of beneficial bacteria, including Bifidobacterium and Lactobacillus, concomitant with a reduction in the levels of detrimental bacteria such as Clostridium perfringens [29]. This modulation of gut microbiota composition was associated with enhanced immune function and a decrease in systemic inflammation, both of which are crucial factors in maintaining overall health during the aging process.

Animal models have provided valuable insights into the effects of LAB on gut microbiota composition. For instance, a study reviewed the impact of LAB derived from traditional fermented Chinese foods on the gut microbiota of aged mice [30]. The findings revealed that LAB supplementation led to a favorable shift in gut microbiota composition, characterized by increased abundance of beneficial bacteria such as Bifidobacterium and Lactobacillus, accompanied by a decrease in harmful bacteria like Clostridium perfringens [31].

The supplementation of LAB represents a promising avenue for modulating the composition of the gut microbiota and restoring gut homeostasis in the aging population[32]. By selectively enriching the abundance of beneficial bacteria and suppressing the growth of harmful species, LAB supplementation holds significant potential for mitigating age-related dysbiosis and promoting overall health in elderly individuals. However, further research is warranted to elucidate the specific mechanisms underlying LAB-gut microbiota interactions and to optimize the application of LAB-based interventions for the prevention and treatment of age-related diseases.

### 2.3. Enhancing Immune Function and Modulating Inflammation for Healthy Aging

Immunomodulation is a fundamental process that involves modifying the response of the immune system to an antigen. This process can either enhance or suppress immune activity. LAB have been recognized for their ability to enhance immune function, which plays a crucial role in healthy aging [33].

In a clinical experiment conducted on healthy individuals above the age of 50 who received an influenza A vaccine, LAB supplementation demonstrated significant effects on the immune response. Specifically, it led to an increase in the production of cytokines and chemokines, which are vital components of the immune system's response to infection and inflammation [34]. Cytokines are proteins produced by immune cells and act as messengers that regulate immune responses. They can either promote or inhibit inflammation, depending on the situation. Chemokines, a type of cytokine, serve as chemo-attractants and help recruit immune cells to the site of infection or inflammation, aiding in the appropriate immune response.

In addition to LAB supplementation, targeting the biology of aging with mTOR (mammalian target of rapamycin) inhibitors has shown promise in improving immune function in older adults [35]. mTOR is a protein kinase that plays a crucial role in regulating various cellular processes, including cell growth, metabolism, autophagy, and immune function [36]. By inhibiting mTOR, studies have demonstrated improved immune function in older adults through a reduction in the production of pro-inflammatory cytokines and an increase in the production of anti-inflammatory cytokines [37]. This is particularly important because as individuals age, their immune system becomes less efficient at responding to antigens, making them more susceptible to infections [38, 39].

Notably, LAB consumption has been associated with potential immune-boosting effects [40, 41]. A study evaluated whether the intake of yogurt fermented with Lactobacillus delbrueckii ssp. bulgaricus (L. bulgaricus) OLL1073R-1 has an effect on resistance to the common cold [42]. A meta-analysis of the results of this study showed the risk of catching the common cold was about 2.6 times lower in the yogurt group than in the milk group and the increase of natural killer cell activity was significantly higher in the yogurt group than in the milk group. LAB fermented feed and three types of LAB (L. plantarum, L. acidophilus, B. animalis) can affect the intestinal microbiota and T cell polarization (Th1, Th2, Th17, Treg) in the intestinal lymph nodes and spleens of rats [43].

Another significant benefit of LAB is its ability to exert anti-inflammatory effects. Inflammation is a natural response of the body to injury or infection. However, chronic inflammation can lead to various health issues, including heart disease, diabetes, and cancer [44]. Several studies have suggested that the consumption of LAB-containing foods can contribute to the reduction of inflammation within the body, potentially mitigating the risk of chronic inflammatory conditions [45, 46]. In a mouse model, the use of Food-Grade Lactococcus lactis demonstrated the ability to inhibit allergic responses [47]. This was achieved through a reduction in serum IgE and IL4 levels while increasing the levels of IFN-γ and TGF-β cytokines. These findings indicate an improvement in allergies by promoting Th1 and Treg responses.

LAB supplementation has been associated with increased production of cytokines and chemokines, strengthening the immune response. Additionally, targeting the biology of aging through mTOR inhibition has demonstrated improved immune function in older adults. The consumption of LAB-containing foods has also been linked to immune stimulation and potential anti-inflammatory effects. Nonetheless, more extensive research is necessary to fully understand the mechanisms involved and to ascertain the translatability of these findings to human populations.

### 2.3. Harnessing the Antioxidative Power of LAB: A Promising Strategy for Combating Oxidative Stress in Aging

LAB have emerged as a promising source of antioxidants [48, 49] with the potential to counteract the detrimental effects of oxidative stress, a key factor in the aging process. Extensive research has revealed that LAB produces a diverse array of antioxidants capable of scavenging free radicals and reducing oxidative stress, thereby promoting cellular health and potentially contributing to healthy aging [50, 51]. Free radicals, one of the primary culprits in oxidative stress, are unstable molecules that possess an unpaired electron. To achieve stability, free radicals often snatch electrons from neighboring molecules, triggering a chain reaction that can cause significant damage. Antioxidants produced by LAB, however, act as electron donors, effectively neutralizing free radicals and breaking the chain of damage. This electron donation process helps restore balance within the cellular environment and prevents oxidative stress-induced cellular dysfunction [52, 53]. For instance, Glutathione, a tripeptide composed of cysteine, glycine, and glutamate, is considered one of the most critical antioxidants produced by LAB [54]. It plays a vital role in protecting cells from oxidative stress by directly interacting with ROS and neutralizing their harmful effects. Glutathione acts as a potent free radical scavenger, inhibiting their ability to cause damage to cellular components. LAB also produce other antioxidants, including catalase, superoxide dismutase (SOD), and vitamins C and E, further enhancing their antioxidant capacity [55, 56].

Numerous studies have provided insights into the potential anti-aging effects of LAB. For instance, a study found that administration of LAB exhibited significantly reduced the shortening of telomere and increased the expression of AMPK subunit-α1 [57]. An additional investigation detailed how yogurt and metabolites from Streptococcus thermophilus improved telomere erosion in aging mice induced by D-galactose [58]. The findings shed light on the potential therapeutic applications of these substances in combating age-related cellular deterioration. Moreover, the study provided valuable insights into the molecular pathways involved in telomere maintenance and suggested promising avenues for further research in the field of anti-aging interventions [59]. Additionally, a study published in the Journal of Microbiology and Biotechnology investigated the effects of LAB on oxidative stress within cells [60]. The researchers observed that LAB supplementation increased the activity of antioxidant enzymes, such as catalase and SOD, which are crucial in combating oxidative stress[61]. For example, studies have shown that exopolysaccharides (EPS) and Selenium-enriched exopolysaccharides (Se-EPS) groups derived from Lactococcus lactis subsp. lactis can significantly increase serum and hepatic enzymatic activities, including SOD, catalase (CAT), and glutathione peroxidase (GSH-Px) [62, 63]. By enhancing the cellular defense against ROS, LAB demonstrated the potential to mitigate oxidative damage and contribute to healthier cellular function.

Harnessing the antioxidative power of LAB may open up new avenues for the development of therapeutic strategies, nutraceutical interventions, and skincare products aimed at mitigating the effects of aging. The exploration of LAB-derived antioxidants as natural alternatives to synthetic antioxidants holds promise for enhancing overall well-being and supporting healthy aging in the population. Continued interdisciplinary research and collaboration will undoubtedly contribute to unlocking the full potential of LAB as valuable allies in the quest for healthy aging.

### 2.4. LAB and the Gut-Brain Axis: Promoting Mental Well-being and Cognitive Health in Aging

The gut-brain axis is a complex and essential connection between the gut microbiota and brain function and behavior [64]. It has become increasingly evident that the gut microbiota has a significant influence on brain function by modulating various neurotransmitter systems, including serotoninergic, noradrenergic, dopaminergic, glutamatergic, and GABA-ergic pathways [64-66]. These neurotransmitters play crucial roles in regulating mood, cognition, and overall brain function [67].

Within the gut microbiota, LAB have emerged as one of the key players in this intricate communication with the brain [68, 69]. LAB possess the ability to influence neurotransmitter production, regulate neuroinflammation, and modulate neural pathways involved in stress response and mood regulation [70, 71]. Numerous studies also have indicated a potential link between age-related cognitive decline, neurodegenerative diseases, and LAB [72-75], as well as dysregulation of the gut-brain axis.

Within the realm of scientific investigation into Lactobacillus strains influence on neural and behavioral outcomes, the strain Lactobacillus rhamnosus JB-1 has garnered significant attention [76]. This strain, when administered to mice, has been shown to effectively reduce depressive and anxiety-like behaviors, an effect closely associated with a vagus-dependent mechanism and shifts in cerebral GABAergic activity [77]. Gamma-aminobutyric acid (GABA), a key inhibitory neurotransmitter within the central nervous system, and its receptors are prevalent throughout mammalian organisms [78]. There's a comprehensive corpus of research, underscored by studies such as Wong et al., linking alterations in GABAergic neurotransmission with a spectrum of CNS-related disorders [79]. This spectrum encompasses behavioral anomalies, pain syndromes, sleep disturbances, and perturbations in key functions of the enteric nervous system, including intestinal motility and gastric processes. Venturing beyond the Lactobacillus rhamnosus JB-1 strain, other strains like Lactobacillus brevis (DPC6108) [80], Lactobacillus buchneri (MS) [81], and Lactobacillus paracasei NFRI (7415) [82] have been identified for their capacity to produce GABA. Interestingly, in studies on vagotomized mice exposed to Lactobacillus rhamnosus, only a slight improvement in anxiety and depressive behaviors was observed, devoid of any discernible shifts in GABA receptor expression in the brain[83]. As mentioned above, LAB can modulate brain function by regulating related pathways, in addition to its primary role in neurotransmitter production. For example, other strains of Lactobacillus acidophilus are known to trigger antiviral reactions, especially through TLR2-dependent IFN-β mechanisms in murine-derived dendritic cells [84]. Additionally, certain lactic acid bacterial species have been pinpointed for their role in instigating TLR3-mediated INF-β secretion within intestinal dendritic cells [85].

By administering LAB supplements, it is believed that the balance of the gut microbiota can be restored, leading to positive effects on neurotransmitter systems and reduced neuroinflammation [86]. This, in turn, has the potential to result in improved cognitive function, mood regulation, and overall brain health in aging individuals. Early findings suggest that LAB supplementation may represent a promising strategy for promoting mental well-being and cognitive health during the aging process [87, 88]. However, it is crucial to note that further extensive research is necessary to fully comprehend the mechanisms underlying the effects of LAB on the gut-brain axis. Additionally, determining optimal dosages and formulations tailored to specific age groups or neurodegenerative conditions is of utmost importance. Notably, this intricate connection is modulated not only by LAB but also by a myriad of bacterial species, dietary components, metabolites, and various environmental factors.

## 3. Human Studies: Investigating the Impact of LAB Consumption on Aging-related Parameters

A growing body of research has focused on understanding the effects of LAB consumption on various aging-related parameters in humans. Clinical trials and observational studies have been conducted to evaluate the influence of LAB on skin health, immune function, cognitive function, and gut microbiota composition. These studies provide valuable insights into the potential benefits of LAB in promoting healthy aging.

One study examined the impact of fermented milk containing LAB on cognitive function in middle-aged subjects. The randomized controlled trial involved a group of elderly individuals who consumed fermented milk for a specific duration. The study reported significant improvements in cognitive function, including memory and attention, in the group receiving LAB supplementation compared to the control group [89].

In the realm of skin health, a study investigated natural S-equol supplementation derived from LAB may have a beneficial effect on crow’s feet wrinkles without serious adverse events [90]. The findings revealed that LAB supplementation led to improved skin hydration and a reduction in the appearance of wrinkles compared to the placebo group.

However, it is important to note that most studies examining the impact of LAB consumption on aging-related parameters have been conducted in animal models or small human studies. Larger-scale human studies are needed to validate these findings and establish more robust conclusions. The limitations of the existing studies should be considered when interpreting the results, as sample sizes may be small, study durations may be short, and confounding factors may not have been adequately controlled.

## 4. Mechanistic Insights into Lactic Acid Bacteria and Longevity: Bridging the Gap

The idea that LAB might influence human health and longevity dates to the observations of Ilya Ilyich Mechnikov, who noted the longevity of Bulgarians consuming large quantities of yogurt [91]. This hypothesis laid the foundation for research into healthful bacteria, known as probiotics, but experimental data on the life-prolonging effects of LAB were lacking.

Recent studies in C. elegans have provided compelling evidence that LAB consumption can indeed increase average lifespan [92]. It is essential to note that while C. elegans is phylogenetically distant from mammals, it serves as a valuable model for investigating molecular mechanisms related to aging. LAB feeding not only extended the nematodes' lifespan but also enhanced their resistance to pathogens, such as Salmonella [93]. This phenomenon is noteworthy, as it suggests that LAB's beneficial effects extend beyond longevity to host defense.

The molecular mechanisms behind the life-prolonging and immunomodulatory effects of LAB are still under investigation. Several mechanisms, including MAPK, IFN-γ, and TGF-β, are implicated in those processes [94, 95]. Of the aforementioned signaling pathways, we postulate that two specific molecules could have a pivotal role in orchestrating a spectrum of responses within the organism, as emphasized in this section.

### Sirtuin-1:

Within the realm of cellular regulation, Sirtuin-1 (SIRT1), the mammalian representative of the Sirtuin family, assumes a pivotal role. Acting as a nicotinamide adenosine dinucleotide (NAD)-dependent deacetylase, SIRT1 targets an extensive spectrum of histone and non-histone proteins, removing their acetyl groups[96]. This enzyme catalytic process not only yields nicotinamide but also segues the acetyl group from the substrate to cleaved NAD, giving rise to the unique metabolite, O-acetyl-ADP ribose [97]. SIRT1 is intrinsically linked to a myriad of physiological endeavors, from gene expression modulation and metabolic regulation to aging processes. Its capability to deacetylate an ever-expanding suite of substrates, which includes but isn't limited to transcription factors such as p53, FoxO family members, and PPARγ [98], underscores its importance. Particularly salient is SIRT1's adeptness at curtailing ROS levels, fostering cellular survival, and offering defense against oxidative stress by manipulating transcription factors, notably the p53 and FOXO families [99]. Its anti-apoptotic attributes are further emphasized by its deacetylation of pivotal proteins, among which are Ku70, PARP, Smad7, and HSF1 [100].

Recent research has delved into the nuanced relationship between SIRT1 and LAB. A seminal study on the probiotic composition, SLAB51, unveiled its profound effect in bolstering SIRT1's expression and activity in the cerebrums of Alzheimer's Disease (AD) mice [101]. In stark contrast, AD mice left untreated manifested an aging-associated surge in acetylation, synonymous with a decline in SIRT1 expression. The integration of SLAB51 led to a pronounced reduction in RARβ-acetylated lysines, a consequence of amplified SIRT1 levels [102]. A mechanism deserving of attention emerges, wherein RARβ activation steers the transcription of the ADAM10 gene, advocating for the nonamyloidogenic APP processing trajectory and simultaneously impeding Aβ peptide synthesis and accumulation [103]. In a quest to spotlight foods and constituents boasting anti-aging potential, a trailblazing system was established, targeting substances that could ignite the SIRT1 promoter in human colorectal cancer cells [104]. A rigorous evaluation of numerous LAB strains resulted in the spotlight being cast on Lactobacillus brevis T2102 for its pronounced SIRT1 activation prowess [105]. Notably, T2102 deterred the proliferation of the DLD-1 human colorectal cancer cell line by deactivating β-catenin through SIRT1-facilitated deacetylation. This cascade also instigated β-catenin degradation, hence inhibiting the transcription of the human telomerase reverse transcriptase (hTERT) gene, a recognized β-catenin target. The upshot was the onset of cellular senescence, paralleled by marked DLD-1 growth inhibition. This discovery positions Lactobacillus brevis T2102 as a potentially transformative entity in sculpting the next generation of functional foods with oncological benefits.

### TLRs

The innate immune system is equipped with germline-encoded pattern recognition receptors (PRRs), among which toll-like receptors (TLRs) are paramount for sensing microbial threats and subsequently orchestrating defensive reactions [106, 107]. Originating from discoveries in the 1990s, TLRs have emerged as specialized PRRs that discern pathogen-associated molecular patterns (PAMPs)—essential entities underpinning microbial pathogenesis, survival, and replication [108, 109]. These PAMPs encompass a vast array of molecules including bacterial toxins, DNA, RNA, and foundational components of cell membranes and walls, spanning across diverse microbial phyla such as viruses, fungi, bacteria, and parasites. Reflecting the vast physiological roles of TLRs, TLR2 expression is omnipresent in a myriad of cells, including immune cells, endothelial cells, and epithelial cells [110]. The TLR family, embedded with evolutionarily conserved leucine-rich repeat motifs, is manifested across a diverse range of cells in numerous animal species. Currently, the spectrum of TLRs is known to comprise 10 human-specific forms (TLR1-10) and 12 variants in mice, with the latter lacking TLR10 [111].

TLR2 merits attention due to its pivotal role in vertebrate immune defense mechanisms. Initially characterized in 1998 alongside its counterparts TLR1, TLR3, TLR4, and TLR5, TLR2 exhibits a unique capability to establish functional heterodimers with an array of TLRs [112]. Moreover, its affinity to engage with a plethora of non-TLR entities accentuates its ability to discern a broad spectrum of PAMPs across multiple microbial taxa [113]. An intricate interplay ensues as ligand recognition by TLR2 predominantly stems from its heterodimeric interactions with TLR1, TLR6, or even TLR10 [114]. Crystallographic insights have delineated the architectural blueprints of these heterodimers, revealing an "m" shaped liaison with ligands. The foundational LRR motifs bestow a horseshoe contour to the ectodomain, further stabilizing the heterodimer. Emerging studies allude to potential TLR2/TLR10 dimer formations, although their biological implications remain to be elucidated [115]. Amid the plethora of ligands that can engage with TLR2, recent evidence postulates that only lipoproteins/lipopeptides qualify as authentic TLR2 ligands, despite the gamut of diacyl, triacylglycerol entities, proteins, and polysaccharides previously considered [116]. Activation of TLR2 by these ligands typically galvanizes a MyD88-centric signaling pathway, a paradigm shared by all TLRs except TLR3 [117]. This signaling conduit facilitates the translocation of nuclear factor-B (NF-B) into the nucleus, modulating gene transcription dynamics and culminating in the release of inflammatory cytokines. Concurrently, the cascade also instigates serine/threonine-specific protein kinases (MAPKs), modulating both inflammatory gene transcription and mRNA stability through activation protein 1 (AP-1) orchestration.

LAB is integral to intestinal health, playing a crucial role in enhancing the barrier function of gut epithelial cells. This enhancement is particularly pronounced when LABs interact with TLRs, especially TLR2. When examining the effects of LAB on pathways initiated by barrier disruptors like the protein kinase C (PKC) dependent A23187 and the mitogen-activated protein kinase-dependent deoxynivalenol, it becomes evident that only PKC-dependent disruptions are alleviated by TLR2-signalling LAB strains, notably L. rhamnosus CNCM I-4036 [118]. This strain, when in direct contact with immune cells, amplifies the expression of both TLR2 and TLR4. However, its culture supernatants mainly elevate TLR1 and TLR5 expression. While some strains like L. casei CCFM9 and L. reuteri CCFM14 were previously identified as non-inducers of TLR signaling, their culture supernatants were recognized as TLR2 activators. This distinction underscores the differential functionalities of LAB-secreted bioactive elements and the LAB strains. Notably, L. fermentum CCFM787 exerts a bidirectional immunomodulatory effect via both TLR2/TLR1 and TLR2/TLR6 pathways. Nevertheless, its dominant anti-inflammatory response seems to emanate from the stronger stimulation of TLR2/TLR6, rendering its application in actively inflamed conditions unwise.

Further analysis examined the TLR-signaling capacity of bacterial cell-free supernatants (CFS), evaluating NF-κB/AP-1 activation in a reporter line (THP1-XBlueTM-MD2-CD14) against a MyD88-deficient line (THP1-XBlueTM-defMyD). Bar L. reuteri CCFM14 and L. fermentum CCFM787, the majority of bacterial strains communicated solely via the MyD88-dependent pathway. Yet, CFS from these two strains triggered both MyD88-dependent and independent pathways, with the former being dominant. Importantly, the regulatory capabilities of bacterial CFS significantly correspond with their TLR2 signaling activation. This relationship is underpinned by a strong correlation between TLR2 activation and cytokine production in macrophages. Examples include L. fermentum strains (CCFM787, CCFM421, CCFM620) that appear to signal through nucleic acids. In contrast, treatments enhanced TLR2 activation in CFS from L. plantarum and L. casei strains. Intriguingly, bacterial CFS manifested M1-polarizing tendencies, suggesting their possible utility in augmenting immunity against malignancies and infections, given the established link between M1-polarized macrophages and suppression of tumors and infections.

Overall, it becomes clear that the TLR family, particularly highlighted by TLR2, serves as a key molecular player during the early stages of cellular responses to bacteria, their metabolites, apoptotic derivatives, and other bacterial components. They play an indispensable role in identification and attachment, directing signals internally and subsequently activating pivotal molecules such as IRF3, NF-κB, and MAPKs. This activation cascade results in the induction of type I IFN and genes coding for inflammatory cytokines. In juxtaposition, the Sirtuin family, exemplified notably by Sirtuin-1, undertakes diverse responsibilities when cells are activated by bacterial components and derivatives. For instance, they play a role in sparking the activation of crucial entities like P53, the FoxO family, PPARγ, and SMAD, each central to senescence-linked pathways. Notwithstanding the expansive studies on LAB with aging, which have shed greater light than in previous times, the intricate interplay between them is yet to be fully grasped. Ambiguities persist regarding which elements, under specific circumstances, can harness TLR2's potential for anti-aging benefits. Similarly, determining the exact component and pathway through which sirtuin-1 achieves its anticipated anti-aging effects remains unresolved. Consequently, a more rigorous exploration is imperative to fully unveil the nexus between LAB and the mechanisms of anti-aging.

## 5. Safety Considerations: Adverse Effects and Medication Interactions

When considering LAB consumption, it is essential to address safety concerns associated with their use. In general, LAB products are considered safe for consumption, and adverse effects are rare. However, some individuals may experience mild gastrointestinal side effects such as bloating, gas, or diarrhea when consuming LAB products [119]. These side effects are generally temporary and resolve on their own.

It is important to note that LAB supplements can potentially interact with certain medications, such as antibiotics and immunosuppressants [120]. The interaction between LAB and medications can affect the efficacy or safety of the drugs. Therefore, individuals taking medications should consult with their healthcare providers before starting LAB supplementation to ensure there are no potential interactions or adverse effects.

Human studies investigating the effects of LAB consumption on aging-related parameters have shown promising results, indicating potential benefits in areas such as cognitive function and skin health. However, further research is needed, particularly larger-scale clinical trials, to validate these findings and establish the optimal dosage and duration of LAB supplementation. Safety considerations related to LAB consumption include the possibility of mild gastrointestinal side effects and potential interactions with certain medications or even food [121]. Adhering to laboratory safety regulations is crucial to ensure the safe handling of LAB and other biological materials in research and testing settings.

## 6. Future Perspectives and Conclusion

LAB are favorable microorganisms extensively employed in food fermentation and as probiotic supplements. They confer a range of health advantages, from enhancing digestive functions and fortifying immune responses to attenuating inflammation. Emerging research posits that LAB may play a role in anti-aging by influencing critical molecular pathways implicated in aging, such as IL-13, TNF-α, Mtor, IFN-γ, TGF-β, AMPK, and GABA. Notably, the TLR family, with an emphasis on TLR2, is central during initial cellular reactions to bacteria, their derived metabolites, and other bacterial elements. Meanwhile, the sirtuin lineage, chiefly represented by Sirtuin-1, assumes varied roles during cellular activation due to bacterial elements and their products. These postulated anti-aging outcomes encompass the re-establishment of gut equilibrium, augmented antioxidant efficacy, and the bolstering of mental acuity and cognitive robustness. Nevertheless, the empirical basis for these assertions remains in its nascent stages and mandates more comprehensive human studies and investigative mechanistic exploration. Additionally, careful appraisals of the safety and ideal intake parameters for LAB are paramount. Prospective inquiries should pivot towards pinpointing specific LAB strains and their metabolites with pronounced anti-aging capabilities, while also demystifying the precise biological conduits facilitating their effects. Given their potential, LAB may emerge as a viable dietary strategy to endorse healthy aging and augment the well-being of the geriatric demographic.
